# Reliability of an interactive sport-specific choice reaction time device

**DOI:** 10.1186/1550-2783-10-S1-P18

**Published:** 2013-12-06

**Authors:** Chih-Yin Tai, Kristy R Crowley, Brandon D Spradley, Laura R Carson, Paul H Falcone, Enrico N Esposito, Michael P Kim, Eric R Serrano, Jordan R Moon

**Affiliations:** 1 Sports Science Center Research Institute, MusclePharm, Inc., Denver, CO, USA; 2 Department of Sports Exercise Science, United States Sports Academy, Daphne, AL, USA; 3 Department of Sport Management, University College of Northern Denmark, Aalborg, Denmark; 4 Department of Human Performance and Exercise Science, University of Mobile, Mobile, AL, USA

## Background

The purpose of this study was to establish the reliability of an interactive choice reaction testing device (Makoto II Arena) to determine the efficacy of the device as it relates to the field of strength and conditioning and sports nutrition research, as well as to determine what protocols are the most reliable in regards to sports specific movements and time.

## Methods

Twelve recreationally trained males participated in Part *a*, which consisted of two visits (mean +/- SD, 3.7 +/- 1.3 days); a familiarization testing day (V1_a_), followed by a subsequent testing day (V1_b_), and was conducted over a three week investigation period (28 +/- 5 yr, 178 +/- 9 cm, 79.15 +/- 15.7 kg, 17.5 +/- 6.6 % body fat). Part *a* was composed of nine choice reaction time testing protocols, including single step audio (CRA); single step visual (CRV); 15/30s single tower unidirectional [CRS(15s) (30s)]; 15/30s two tower lateral-directional [CRL(15s), (30s)]; 15/30s three tower multi-directional [CRM(15s), (30s)]; and a three tower, 2-minute stick hit test (stick hits). Seventeen recreationally trained males participated in Part *b*, which consisted of two visits (4.9 +/- 1.9 days) following a familiarization day (V1_b_ and V2_b_), and was conducted over a two week investigational period (21.5 +/- 4.7 y, 181.1 +/- 6.1 cm, 85.2 +/- 17 kg, 14.5 +/- 11 % body fat). Part *b* comprised the same choice reaction time testing protocols as Part *a*. Part *c* consisted of a pooled mean of 62 tests taken from Part *a* and Part *b*, which examined data within choice reaction testing days between V1_a_, V2_a_, V1_b_, and V2_b_, except the 2-minute Stick Hits data.

## Results

Mean (+/- SD) time (seconds) values for Part *a*, Part *b*, and Part *c* were 0.87, 0.91 and 0.86 for Day/Trial 1 respectively, and 0.81, 0.89, and 0.85 for Day/Trial 2 which resulted in no significant differences from Day/Trial 1 to Day/Trial 2 for Part *a*, *b*, and *c* (p > 0.05). However, all times between testing days/trials decreased (*a*: -0.071 sec, *b*: -0.021 sec, *c*: -0.010). Differences in days from Part *b* (-0.02 sec) and Trials for Part *c* (-0.01 sec) resulted in similar findings, suggesting a familiarization session between testing days may result in similar reliability to that of within-day trials (p = 1.00). Two testing batteries showed a significant decrease in time between Day 1 and Day 2 after familiarization: CRL15 (Mean difference = -0.07, p = 0.036) and CRM30 (Mean difference = -0.05, p = 0.022).

## Conclusions

A day of familiarization improved the reliability of all tests. Single step, 30 second, and 15 second tests appear to be reliable. Furthermore, the current study suggests that a “predominantly” upper body unidirectional choice reaction test lasting 30 seconds may be more reliable than a test which utilizes multi-joint or multi-direction functioning lasting 15 seconds or less, however, the reliability within and between days appears to be no different for the tests used in the current investigation suggesting the device and methods used in the current investigation are acceptable for use in strength and conditioning and sports nutrition research.

